# The experiences of patients with multiple sclerosis of self-compassion: A qualitative content analysis

**DOI:** 10.37796/2211-8039.1211

**Published:** 2021-12-01

**Authors:** Hanie Dahmardeh, Afsaneh Sadooghiasl, Eesa Mohammadi, Anoshirvan Kazemnejad

**Affiliations:** a Ph.D. Student, Department of Nursing, Faculty of Medical Sciences, Tarbiat Modares University, Tehran, Iran; b Department of Nursing, Faculty of Medical Sciences, Tarbiat Modares University, Tehran, Iran; c Department of Biostatistics, Faculty of Medical Sciences, Tarbiat Modares University, Tehran, Iran

**Keywords:** Self-compassion, Multiple sclerosis, Qualitative content analysis

## Abstract

**Background:**

Self-compassion enhances self-care behavior in patients with multiple sclerosis. This concept has been defined in previous studies; however, in order to effectively enhance it, patients’ perceptions about and experiences with self-compassion should be first understood. Therefore, this study aims to explore the meaning of self-compassion experienced by patients with multiple sclerosis.

**Methods:**

This directed qualitative study was conducted in 2019 in Iran. Twenty-three patients with Multiple sclerosis were selected purposefully and interviewed individually. Qualitative content analysis was used for data analysis according to Hsieh and Shannon’s method.

**Results:**

Seventy-six primary codes were detected as well as the following eleven categories: self-kindness, self-judgment, common humanity, isolation, mindfulness, over-identification, seeking support, concealment, spiritual resilience, marital life concern, and turning into an example for others. These categories express the characteristics and meaning of self-compassion in patients with multiple sclerosis. Six of the 11 characteristics are in accordance with the theory of Neff’s theory and five are related to patients and the cultural and social arena of the study environment.

**Conclusion:**

Results of the present study showed that new dimensions of self-compassion were found by exploring multiple sclerosis patients’ experiences, which added to the suggested dimensions of others. This study is promising to nurses and paramedics as it will help them to better identify and to take better care of patients. The result will also help to design a valid tool to measure this issue of patients.

## 1. Background

Multiple sclerosis (MS) is a chronic, inflammatory, progressive and common autoimmune disease of the nervous system [1,2]. Most people suffer from it at a young age, around their 20s–40s. With many debilitating symptoms and complications [2], it is the most common cause of neurological disability in adults [3].

Considering the features of this disease, and as the number of individuals suffering from MS is increasing [2], concerns have been raised regarding the quality of life and healthcare expenses of these patients. The impact and effectiveness of self-care have been observed before in patients’ medical and personal outcomes [4]. Self-care can reduce patients’ expenses, improve their quality of life, and it should be noted that its significance has been evident in patients’ medical and person-centered results [5]. The concept of self-compassion has a key role in self-care [6].

Neff (2003/2009) was the first person to discuss the dimensions of self-compassion [7–10] and divided them into three primary categories (e.i. common humanity, mindfulness, and self-kindness) and three subcategories including over-identifying, self-judgment, and feeling isolated [11,12].

It should be noted that self-compassion is affected by the values, beliefs, and culture of the society [13]. Also, self-compassion and its relation to self-care [7], especially in MS patients, is important as these patients usually suffer from this disease when they are young and cannot perform effectively in society [14]. Therefore, further studies are needed to understand the dimensions of self-compassion in different communities. This study was aimed at exploring the meaning and dimensions of self-compassion in Iranian MS patients; and based on the researcher’s knowledge, no study has been conducted on this issue before.

## 2. Methods

### 2.1. Aim

This study was aimed at exploring the meaning of self-compassion from Iranian MS patients’ perspectives.

### 2.2. Design

Directed qualitative content analysis [15] was used alongside Neff’s definition of self-compassion, and all previously suggested dimensions of self-compassion proposed by Neff and other scholars, to better understand MS patients’ perception of self-compassion.

### 2.3. Sample and setting

Participants were purposefully selected from MS patients who had been diagnosed with the condition, from whose diagnoses at least 6 months had passed, and they had received at least 6 months of treatment for it. The setting of the study was hospitals and communities of Zahedan and Tehran Province, Iran. Table 1 shows the characteristics of the participants (Table 1).

After obtaining informed consent, interviews were carried out until data saturation was met. In general, 23 interviews were carried out and from the 18th interview onwards, no new codes were discovered.

### 2.4. Data collection

Individual, semi-structured, in-depth face-to-face interviews were used for data gathering. Questions were designed based on Neff’s dimensions of self-compassion. The interviews lasted from 15 to 55 minutes and the average duration of each interview was 28 minutes. Participants were informed about the aim of study, with whose consent the interviews were carried out at a time and location of their own choosing – the participant’s comfortability was considered all through the interview. All interviews were conducted and recorded in Persian by the first author, some questions of which are provided hereunder:

1. From the moment you were diagnosed with MS how have you been feeling about yourself?
2. Have you ever thought about the cause of this problem? And how have you evaluated it?
3. How do you feel about yourself when you think about or see other people suffering from MS as well?

### 2.5. Data analysis

Interviews were immediately transcribed word for word, and read several times to ensure a deep common understanding of the participant’s statements. Data were analyzed using Hsieh and Shannon’s (2005) content analysis has three stages of preparation, organization and reporting [15]. In the preparation phase transcribing the interview, each text should be read several times until data is immersed. Then, in the organization stage, the researcher designs an unconstrained matrix that would allow the detection of the main concepts – or in other words, the main categories. In order to find the content that corresponds or could have corresponded to the previously defined categories, data is reviewed many times and primary codes are assigned. Here, other meaningful units are encoded which are unrelated to the main categories but related to the concept of self-compassion in general, based on the conceptual and logical relevance of which, the possibility of incorporating these categories into existing main categories of the matrix and/or the formation of new main categories is explored [16]. All the above was carried out step-by-step in this study and gathered data was analyzed by use of MAXQDA software version 10. Table 2 illustrates this data analysis matrix designed based on Neff’s theory.

### 2.6. Rigor

Lincoln and Guba’s criteria such as credibility, transferability, consistency or dependability, and confirmatory ability were considered in this study. Member and peer checking as well as prolonged engagement techniques were used. Participants were selected with maximum variation in age, gender, marital status, educational status, length of disease, severity of disease, and disease complications. After encoding the text of an interview, it was returned to the participant to ensure that both the interviewer and interviewee had the same understanding of it. Data analysis and encoding were supervised and confirmed by the research team (the second and third authors) who are well experienced in performing qualitative research and investigating MS patients as well as issues like self-compassion. The researchers documented all stages of this research, including data gathering, analysis, and encoding (into categories and subcategories) so that it would be explored by others.

### 2.7. Ethical considerations

This study has been approved by Institutional Review Board (IRB) of Tarbiat Modares University, Medical School with number: IR.MODARES. REC.1398.139. All participants completed written Informed consent. COREQ checklist was used to report the study. All procedures performed in studies involving human participants were in accordance with the ethical standards of the institutional and/or national research committee and with the 1964 Helsinki declaration and its later amendments or comparable ethical standards.

## 3. Results

Seventy-six primary codes were extracted. Some of these codes were placed in Neff’s suggested six categories and Freeman’s “seeking support”, while the rest were placed in the following four new categories: spiritual resilience, concern for marital life, turning into an example for others, and concealment (Table 2).

### 1. Self-kindness

According to Neff’s theory, self-kindness means supporting self with kindness and understanding instead of judging or reproaching self when one is in distress [11,12].

In this study, MS patients have associated it with issues like their diets, medication, self-care, self-esteem and self-worthiness.

“When I learned that I had this disease and I received the doctor’s final diagnosis, I began searching and learning about it, even on the internet, and checked to see what would make it worse or what would make it better, and what would help to control it” (p^1^8)

### 2. Self-judgment

Based on Neff’s theory, self-judgment means judging and reproaching oneself when there is pain and distress, which is the opposite of self-compassion [11,12]. In this study, MS patients have referred to it as “not caring for oneself” and “blaming self for getting ill”. These patients shared how they had become MS patients in the first place, how they have been preoccupied with the disease ever since, and how the thought had troubled them.

“I wasn’t thinking about myself at all, I was concerned about my little baby … It wasn’t such a big deal for me to cry or snob, I was more worried about my little baby” (p2).

### 3. Common humanity

Based on Neff’s theory, “common humanity” stands for perceiving one’s experiences as part of the larger human experience rather than perceiving them as experiences that would isolate them from others [11,12]. In this study, MS patients believed that it can be achieved by fostering a positive spirit in oneself and attempting to contribute or carry out responsibilities like they used to before getting sick.

“I like to be an independent individual myself, I have come here and been hospitalized, I haven’t allowed anyone to visit me like the way they visit others, or asked anyone to bring tea or to do something for me, I tell them “no” and that I can do my stuff by myself. I am an independent person and am fine”. (p 16)

### 4. Isolation

Based on Neff’s theory, “isolation” means finding oneself the only one having the disease and alone in it; this is the opposite of “common humanity” [11,12]. In this study, MS patients have described it by terms like distance, loneliness, having negative thoughts about self, and becoming dissociable.

“I am mostly depressed, I can’t stand others I don’t feel like having people over nor do I feel like going anywhere. I just stay there alone.” (p 17)

### 5. Mindfulness

Based on Neff’s theory, in the state of “mindfulness” a person observes his/her feelings and experiences as they are without magnifying them [11,12]. In this study, MS patients have associated “mindfulness “ with other implications like “trying to forget being ill”, “trying to avoid thinking about the disease and its negative impacts”, “becoming adaptable and accepting the difficult treatment”, “self-construction and turning the bad feeling into good ones”, “changing one’s perspective about the disease”, “accepting the disease and coping with it” and “not blaming self because of the disease”.

“I was think it would be worse, so I didn’t think about it at all … I didn’t think negatively that much as in *Hey, you are going to become paralyzed or something* ….”. (p14)

### 6. Over-identification

Based on Neff’s theory, “over-identification” happens when patients magnify problems in their minds, which is the opposite of mindfulness [11,12]. In this study, in addition to the terms mentioned above, MS patients have associated “over-identification” with implications like “a paralyzed mentality” and “severe inability caused by thinking constantly about the disease and fearing it”, “unwillingness to accept the illness and inability caused by it”, “concerns about the progress of the disease by observing other patients who suffer from more advanced conditions. They expressed their concern about the progress of the disease when they encounter or had encountered patients with advanced conditions.

“I was so hopeless that I kept saying that I will die just like my sister and cousin, and I had this terrible hopeless feeling. I kept saying that I would be left alone, I would become paralyzed, I would go blind, I would get really worse … I thought about not being able to do my chores and had this really bad feeling. I kept saying I would become disabled and would need others to do my stuff … (p 22)

### 7. Seeking support

Freeman’s (2016)” seeking support” is when MS patients ask others for support [17]. In this study, MS patients described it as a “positive perspective”, “encouragement” and “supporting behaviors” that they had observed in their family members and physicians.

“My family respects me a lot and really considers my condition. The thing that MS patients hate is when people feel sorry for them. But I myself like this sympathy because I know that they love me and this is why they are doing my stuff. And this is why it didn’t feel bad, my family supported me a lot … this made me feel much better and this is why I adored their support” (p12)

With reference to the experiences of the participants, a few new categories were found as follows:

### 8. Concealment

MS patients describe it as hiding the disease from others to avoid their possible sympathy which would upset the patient or cause discomfort. Additionally, patients have also associated concealing with issues like caring for oneself and not having expectations from others.

“Sometimes someone might say something to me and try to put their kindness in words but the problem is that it is coming from their sense of sympathy, I mean they feel sorry for me, and I don’t like it”. (p9)

### 9. Spiritual resilience

MS patients associate spiritual resilience with thanking God that their illness was diagnosed early, that their illness was treatable and they had gotten better; additionally, performing religious activities, being happy and feeling fresh as the result of relying on God and believing that everything is in His will and command.

“And I thank God that I learned that I have this disease soon enough, I mean I didn’t let a year or two or more to pass …” (p2)

(p4)

### 10. Marital life concern

MS patients believe that the disease is an obstacle that is preventing them from getting married.

“I’ve seen the people around me, at my age and married, some even have kids but I have none. These moments make me regret that I didn’t get married while I was 18, I could have even had kids by now …”(p11)

### 11. Turning into an example for others

MS patients tried to share their positive experiences with others and turn into an example or a guide for others.

“After a while that I was hospitalized here, I saw this patient … He was in a very bad condition … I tried to keep his spirit high and told him that he shouldn’t be like this – because he was getting really worse and he had lost all hope. I told him a lot of these stuff like he shouldn’t lose his hope …”(p15)

## 4. Discussion

The purpose of this study was to explore the meaning of self-compassion in MS patients. Our findings repeated previously suggested dimensions of self-compassion like self-kindness, self-judgment, common humanity, isolation, mindfulness, over-identification [11], and seeking support [17], and added four new dimensions of concealment, spiritual resilience, concern about marital life, and turning into an example for others.

Findings were in concordance with the results of Klingle (2017) who explored the perception of self-compassion among juveniles with difficult and painful life experiences. Kingle’s two implications of “Putting oneself in the center of attention” and having “a balanced emotional experience” are similar to “becoming attractive to others” and “mindfulness” in this study, respectively. Also, Reekers’ (2012) study which was conducted on social workers showed that dimensions like “gentleness”, “mindfulness” and “human connection” overlap with dimensions like “self-kindness”, “mindfulness” and “common humanity”, respectively [18]. Dimensions like “connecting with the experiences of others”, “recognizing distress”, “identifying personal accountability” and “allowing the agency of others” suggested in Freeman’s (2016) study conducted on teachers, overlap with “humanity” and “mindfulness”, respectively [17].

The results of this study are similar to those of the abovementioned studies as self-compassion is a natural, acquiring and multidimensional feature of a human [19]. Various factors such as life experiences, cultural conditions, and values [20] are effective in people’s understanding of self-compassion but compassion, satisfaction, love patience, and tolerance are all features of human nature [21]. In this study, MS patients who have chronic conditions inherently have human nature and features despite the different socio-cultural settings that they are in or the various life experiences that they have, and Neff’s six dimensions are also applicable to them.

Concealment is a new dimension used to explore self-compassion in this study and it means that the patient tries to rely on him/herself and not seek support from others by concealing his/her disease. This dimension is the opposite of seeking support [17] which meant that one seeks the support of others during one’s illness. Acorn and Joachim (2000) showed that patients who have chronic illnesses with observable or unobservable symptoms may conceal their illness from others to avoid sympathy, stigma, and/or isolation. Also, MS patients too have observable and unobservable symptoms and the same issue applies to them as well [22].

Another finding of this study was religious resilience. MS patients try to tolerate or overcome their illness by patience, trusting in God’s will, and also performing religious activities.

Resilience is a kind of adaptation or strengthening that one has when encountering difficulties, which is achieved by interior and exterior resources. On one hand, spirituality is a way to reach adaptability and a method to facilitate the understanding of issues or intentions. Spirituality and resilience are interrelated and spiritual resilience is an ability by which one can maintain one’s feelings and intentions by a body of beliefs, values or principles. This is while, by use of interior and exterior spiritual resources, one may encounter stress, pressure and harm [23]. In Rickers’ study (2012) acceptance is a dimension which stands for accepting one’s strengths and weaknesses as well as accepting specific challenging conditions which the patient may also have no control over; and it also stands for calmness when individuals experience self-compassion [18]. In this study, MS patients by accepting their illness, by relying on God, and by being patient and calm, try to carry out their religious deeds and try to reinforce their positivity by thinking of God and accepting His will.

Pham et al.’s (2019) study on patients with chronic kidney disease showed that the meaning of transcendence, religion, church attendance, prayer and other spiritual activities, make individuals stronger and more able to reinforce their positivity, relying on self, connecting to others and God, and empowering oneself to be ready to live with a chronic disease [24].

Over 97% of the participants of this study are Muslims [25], so their beliefs and values influence their lives and behaviors [26]. In Islamic texts, dimensions like considering one’s bounty and happiness, avoiding sorrow, coping with self, having compassion, avoiding negligence and being fair about self, are suggested dimensions for self-compassion [27]. In this study, MS patients try to tolerate their illness by relying on spiritual activities, religion and resilience.

Another new category was being concerned about one’s marital life which was extracted from the experiences that the participants shared while explaining how they perceived self-compassion. Married patients were worried that their marriage might end, and single patients were concerned that they may never get married. When physical health is impaired in a chronic disease, it significantly affects one’s interactions with one’s partner and relatives [28].Living with a chronic progressive disorder such as MS not only causes stress for patients but also brings considerable anxiety to the patient’s dear ones. This disease affects the patient’s familial and social life, and patient may suffer from family disputes, social conflicts, divorce, etc. [29].This disease is complicated and threatening which brings a great deal of stress for one’s spouse and may lead to an unhappy marriage, separation or divorce. The results of different studies have shown that the rate of divorce is high in families where one partner is suffering from MS [30]. As expected, in this study MS patients were concerned about their marital life.

Becoming a model is another category which was extracted from the findings of this study regarding MS patients. It implied working hard to transfer one’s positive experiences and vibes to other patients and becoming their somewhat sponsor. Denis (2003) defines ‘peer support’ in the context of healthcare as: “providing emotional support, evaluating, and informing which are done by a member of a social network that has empirical knowledge of a certain behavior or factors that cause stress and similar issues to the target population” [31].

In Freeman’s (2016) studies, relating positively with others’ experiences is a dimension of self-compassion; and this study has also shown the same. Here, MS patients try to help other patients who are suffering from a similar condition to overcome or tolerate the disease, by sharing their own relevant positive experiences. This would help others to better understand the challenges that they encounter, while simultaneously express self-compassion towards themselves [32].

## 5. Conclusion

Given that Neff’s study was conducted on healthy individuals who had not suffered from difficult conditions, the dimensions that were found in this study did not surface in Neff’s. For MS patients however, who have suffered challenging conditions and have different experiences, self-compassion has a different range of dimensions. The study results can be used for improving clinical patient care, promoting self-care among patients with Multiple Sclerosis, and to design a valid tool to measure this issue.

## Supplementary Information







## Figures and Tables

**Table 1:** Characteristics of study participants.

Different levels of severity of disease and its complications		Duration of illness (year)		Educational status		Marital status		Gender	
				
Variable	Data	Variable	Data	Variable	Data	Variable	Data	Variable	Data
Eye problems (poor eyesight)	12	Six months-1 year	2	Illiterate	1	Single	7	Female	20
Difficult walking (walking disorder)	13	2–5 years	6	High school	6	Married	13	Male	3
Urinary and bowel problem	4	6–10 years	10	Diploma	6	Divorced	3		
Fatigue	23	11–15 years	4	Bachelor’s degree	9				
Limb paralysis	2	16–20 years	1	Master’s Degree	1				
Muscle Weakness	13								

**Table 2:** Data analysis matrix based on Neff’s dimension.

Categories based on Neff’s dimension	Primary codes
Self-kindness	Carrying for the proper consumption of medicine, despite the fact that it one might not consider it to be exclusory Relying on medication and proper nutrition for self and family Relying on medication for one’s family Following-up on the treatment Feeling more confident at home than in society Feeling more confident and valuable Emphasizing confidence, self-esteem, optimality and empowerment Paying more attention to one’s health and self-care Trying to avoid stressful and uncomfortable situations Putting oneself at the center of attention Reaching a sense of self-love and self-respect
Self-judgment	Not respecting self Caring more for others and less for self Not taking one’s illness seriously Blaming self and one’s former behavior for getting ill Feeling insignificant to others Mindfulness (an occupied mind) about why one is now suffering from the illness
Common humanity	Trying to induce the feeling of hope to for more effective treatment and reinforcing positivity in self Trying to show oneself positive and normal Trying to develop a positive perspective towards the weaknesses that have been caused Trying to rely on self Emphasizing patience and tolerance to overcome the illness Trying to perform social roles (studying and exercising) just like one did before getting ill Trying to represent self like a healthy person Developing a good feeling/hope in self by observing/speaking with other patients who suffer from the same illness Developing a feeling that one is good and better when one compares one’s condition with that of other patients
Isolation	Avoiding others Having negative thoughts about self and becoming isolated Willing to be distant and alone
Categories based on Neff’s dimension	Primary codes
Mindfulness	Trying to forget and not think about the illness Concealing the illness from one’s family to maintain one’s health and comfort Not believing that the illness is an obstacle to living Avoiding thinking about the illness and its negative aspects Accepting the illness and coping with it Accepting the illness and at the same time relying on God Not blaming self about for the illness Observing the illness as a simple issue Trying to overcome symptoms that affect the senses, though simple ways Emphasizing optimism and proudness when the symptoms of the illness no longer reappear Emphasizing the effort to overcome the symptoms that negatively affect the illness Not feeling bad because of not knowing the nature of the illness Converting bad feelings about the illness to good ones Changing one’s perspective towards the illness
Over-identification	Doing less activities because of the illness To attribute the emergence of new plaques to their coping Not willing to accept the illness and the inability that follows The thought and concern about the progress of the illness by observing other patients suffering from advanced stages of the illness Fear of paralysis Thinking a lot about paralysis Mental paralysis and severe inability as the result of the illness
Seeking support	Being happy about what one has and being thankful for one’s family support Reaching a feeling of calmness when there is family and doctor’s support, trying to be positive and perform effectively Being positive about the consideration, thoughtfulness and support of others Feeling better and more positive with the support, words, or help of the doctor

Remaining codes that could not be	Primary code

New category	

Concealment (the negative dimension of support seeking)	Concealing the illness from others to avoid others’ pity Not wanting or needing the pity of family or others Not wanting others’ attention Being upset if others perform one’s tasks Thinking about self and not expecting from others
Religious resilience	Understanding and being thankful that the illness is treatable Thanking God that one’s condition is getting better Thanking God that that the illness is diagnosed Thanking God when comparing one’s condition with that of others whose condition is worse Reinforcing calmness and positivism with believing in the illness Knowing that the illness is a God’s way of testing one Becoming calm when performing religious activities Not committing suicide for fearing God Feeling happy and fresh when relying on God Emphasizing on the religious explanation of becoming ill, and being patient
Concern about martial life	Concern about not getting married Concern about the future of a martial life
Becoming a role model for others	Trying to become a model or guide for others while also carrying for one’s health Becoming a model for others Transferring one’s positive feelings and experiences to others

## Footnotes

1 Patient.

## References

[1] Abedini E Ghanbari Hashem Abadi BA Talebian-Sharif J Effectiveness of group therapy based on hope approach on hope and depression in women with multiple sclerosis J Clin Psychol 2016 8 2(30) 1 11

[2] Dahmardeh H Vagharseyyedin SA Amiri Fard H Sharifzadeh GR Rakhshani-Zabol F Effect of self-care educational program based on Orem’s Theory on hope in patients with Multiple Sclerosis Med - Surg Nurs J 2015 4 2 57 63

[3] Karussis D The diagnosis of multiple sclerosis and the various related demyelinating syndromes: a critical review J Autoimmun 2014 48 49 134 42 24524923 10.1016/j.jaut.2014.01.022

[4] Jaarsma T Cameron J Riegel B Stromberg A Factors related to self-care in heart failure patients according to the middle-range theory of self-care of chronic illness: a literature update Curr Heart Fail Rep 2017 14 71 7 28213768 10.1007/s11897-017-0324-1 PMC5357484

[5] Wilkinson A Whitehead L Evolution of the concept of self-care and implications for nurses: a literature review Int J Nurs Stud 2009 46 1143 7 19200992 10.1016/j.ijnurstu.2008.12.011

[6] Sinclair S Kondejewski J Raffin-Bouchal SM King-Shier K Singh P Can self-compassion promote healthcare provider well-being and compassionate care to others? Results of a systematic review Appl Psychol: Health Well-being 2017 1 39 10.1111/aphw.12086 28393485

[7] Ferrari M Dal Cin M Steele M Educational and psychological aspects Self-compassion is associated with optimum self care behaviour, medical outcomes and psychological well-being in a cross-sectional sample of adults with diabetes Diabet Med 2017 34 11 10.1111/dme.13451 28799282

[8] Nery-Hurwit M Yun J Ebbeck V Examining the roles of self-compassion and resilience on health-related quality of life for individuals with Multiple Sclerosis Disabil Health J 2017 1 18 29089214 10.1016/j.dhjo.2017.10.010

[9] Amanullah A Tirdat K Aslani K Prediction of depression based on components of self-compassion in girl students with emotional breakdown experience in Ahvaz Universities J Clin Psychol 2016 8 2 77 87

[10] Moszadeh S Effectiveness of self-directed group training on reducing the stress of parenting and increasing parental self-efficacy and resilience of mothers of children with autism spectrum disorder Mashhad Ferdowsi University 2017

[11] Neff KD Self-compassion: an alternative conceptualization of a HealthyAttitudeTowardOneself Self Ident 2003 2 85 101

[12] Raes F Pommier E Neff KD Van Gucht D Construction and factorial validation of a short form of the self-compassion scale Clin Psychol Psychother 2011 18 250 5 21584907 10.1002/cpp.702

[13] Neff KD Pisitsungkagarn K Hsieh Y Self-compassion and self-construal in the United States, Thailand, and taiwan J Cross Cult Psychol 2008 39 3 267 85

[14] Reyes D Self-Compassion:A concept analysis J Holist Nurs Am Holist Nurse Asso 2012 30 2 81 9 10.1177/0898010111423421 22024954

[15] Hsieh HFSS Three approach to qualitative content analysis Qual Health Res 2005 15 9 1277 88 16204405 10.1177/1049732305276687

[16] Elo S Kyngas H The qualitative content analysis process J Adv Nurs 2008 62 1 107 15 18352969 10.1111/j.1365-2648.2007.04569.x

[17] Freeman S Emotions in teaching: self-compassion Brigham Young University 2016

[18] Rickers S The lived experience of self-compassion in social workers The university of minnesota 2012

[19] Montero-Marin J Kuyken W Crane C Gu J Baer RA Al-Awamleh A Self-compassion and cultural values: a cross-cultural study of self-compassion using a multitrait-multimethod (MTMM) analytical procedure Front Psychol 2018 9 10.3389/fpsyg.2018.02638 PMC6308155 30622499

[20] McGehee P Germer C Neff KD Core values in mindful self-compassion Practitioner’s guide to ethics and mindfulness- based interventions 2017 279 93 10.1007/978-3-319-64924-5_11

[21] Neff KD Rude SS Kirkpatrick K An examination of self compassion in relation to positive psychological functioning and personality traits J Res Pers 2007 41 4 909

[22] Joachim G Acorn S Stigma of visible and invisible chronic conditions J Adv Nurs 2000 32 1 243 8 10.1046/j.1365-2648.2000.01466.x. 10886457

[23] Manning L Ferris M Rosario CN Prues M Bouchard L Spiritual resilience: understanding the protection and promotion of well-being in the later life J Relig Spiritual Aging 2018 1 20 10.1080/15528030.2018.1532859 PMC7743140 33335455

[24] Tony VP Cherry MB Jane PG Harold GK John WS Spirituality, coping, and resilience among rural residents living with chronic kidney disease J Relig Health 2019 1 18 31392626 10.1007/s10943-019-00892-w

[25] Fathi E A look at the religion and population of Iran in the half a century Statistics 2016 21

[26] Yaghob F Demographic survey of Muslims in contemporary world Islam World Polit Res Quarterly 2015 3 5 61 98

[27] Zinali R Compassion for oneself in Islamic teachings mishkat 2016 132 Fall 38 60

[28] Alavian SM Kachuee H Moghani Lankarani M Assari S Farmanara H Marital adjustment in patients with chronic viral hepatitis versus healthy controls Iran J Psychiatry 2006 1 4 103 7

[29] Sadat SJ Alimohammadi N Alamdary AK Phenomenological study of family relationships and Social patients with multiple sclerosis J Mazandaran Univ Med Sci 2011 21 1 244 202

[30] Tajikesmaeili A Hakim abadi MG Sexual functions and marital adjustment married woman with Multiple Sclerosis J Res Psychol Health 2016 10 2 1 10

[31] Dennis CL Peer support within a health care context: a concept analysis Int J Nurs Stud 2003 40 321 32 12605954 10.1016/s0020-7489(02)00092-5

[32] Ng L Amatya B Khan F Outcomes of a peer support program in multiple sclerosis in an Australian community cohort: a prospective study J Neurodegenerat Disease 2013 1 1 7 10.1155/2013/429171. PMC4437345 26316989

