# Supplementary material for: The experiences of patients with multiple sclerosis of self-compassion: A qualitative content analysis
Source: Biomedicine (Taipei). 2021 Dec 1;11(4):35–42. doi: 10.37796/2211-8039.1211 (PMC8823481; doi:10.37796/2211-8039.1211)
Supplement: Supplementary file 1 [file bmed-11-04-035-s001.docx]

**Abstract**

**Background:** Self-compassion enhances self-care behavior in patients with multiple sclerosis. This concept has been defined in previous studies; however in order to effectively enhance it, patients’ perception’s about and experiences with self-compassion should be first understood. Therefore, this study aims to explore the meaning of self-compassion experienced by patients with multiple sclerosis.

**Methods:** This qualitative study was conducted in 2019 in Iran. Twenty-three patients were selected purposefully and interviewed individually. Qualitative content analysis was used for data analysis according to Hsieh and Shannon’s method to extend Neff’s self-compassion theory.

**Results:** Seventy-six primary codes were detected as well as the following eleven categories: self-kindness, self-judgment, common humanity, isolation, mindfulness, over-identification, seeking support, concealment, spiritual resilience, marital life concern, and turning into an example for others.

**Conclusion:** Results of the present study showed that, new dimensions of self-compassion were found by exploring multiple sclerosis patients’ experiences, which added to the suggested dimensions of others. This study is promising to nurses and paramedics as it will help them to better identify and address this issue. The results will also help patients to take better care of themselves.

**Keywords:** Self-Compassion, Multiple Sclerosis, Qualitative Content Analysis
