# Supplementary material for: The experiences of patients with multiple sclerosis of self-compassion: A qualitative content analysis
Source: Biomedicine (Taipei). 2021 Dec 1;11(4):35–42. doi: 10.37796/2211-8039.1211 (PMC8823481; doi:10.37796/2211-8039.1211)
Supplement: Supplementary file 2 [file bmed-11-04-035-s002.docx]

**Title page**

**The experiences of patients with multiple sclerosis of self-compassion: A qualitative content analysis**

Hanie Dahmardeh^1^, Afsaneh Sadooghiasl^2^, Eesa Mohammadi^2^, Anoshirvan Kazemnejad^3^

1. Ph.D. Student, Department of Nursing, Faculty of Medical Sciences, Tarbiat Modares University, Tehran, Iran.
2. Department of Nursing, Faculty of Medical Sciences, Tarbiat Modares University, Tehran, Iran
3. Department of Biostatistics, Faculty of Medical Sciences, Tarbiat Modares University, Tehran, Iran.

**Corresponding author:** Afsaneh Sadooghiasl, email: hdah9467@gmail.com

**Tell:** +9821 8288 3145

**Acknowledgements:**

This study is a part of a PhD dissertation by the first author. All authors would like to express their gratitude to Tarbiat Modares University and all participants of this study as well.

**Conflicts of Interest**: The authors have no conflicts of interest to declare.”
