# Supplementary material for: The experiences of patients with multiple sclerosis of self-compassion: A qualitative content analysis
Source: Biomedicine (Taipei). 2021 Dec 1;11(4):35–42. doi: 10.37796/2211-8039.1211 (PMC8823481; doi:10.37796/2211-8039.1211)
Supplement: Supplementary file 3 [file bmed-11-04-035-s003.docx]

**The experiences of patients with multiple sclerosis of self-compassion: A qualitative content analysis**

**Abstract**

**Background:** Self-compassion enhances self-care behavior in patients with multiple sclerosis. This concept has been defined in previous studies; however in order to effectively enhance it, patients’ perception’s about and experiences with self-compassion should be first understood. Therefore, this study aims to explore the meaning of self-compassion experienced by patients with multiple sclerosis.

**Methods:** This qualitative study was conducted in 2019 in Iran. Twenty-three patients were selected purposefully and interviewed individually. Qualitative content analysis was used for data analysis according to Hsieh and Shannon’s method to extend Neff’s self-compassion theory.

**Results:** Seventy-six primary codes were detected as well as the following eleven categories: self-kindness, self-judgment, common humanity, isolation, mindfulness, over-identification, seeking support, concealment, spiritual resilience, marital life concern, and turning into an example for others.

**Conclusion:** Results of the present study showed that, new dimensions of self-compassion were found by exploring multiple sclerosis patients’ experiences, which added to the suggested dimensions of others. This study is promising to nurses and paramedics as it will help them to better identify and address this issue. The results will also help patients to take better care of themselves.

**Keywords:** Self-Compassion, Multiple Sclerosis, Qualitative Content Analysis

**Background**

Multiple sclerosis (MS) is a chronic, inflammatory, progressive and common autoimmune disease of the nervous system [1,2]. Most people suffer from it at a young age, around their 20s to 40s. With many debilitating symptoms and complications[2], it is the most common cause of neurological disability in adults [3].

Considering the features of this disease, and as the number of individuals suffering from MS are increasing [2], concerns have been raised regarding the quality of life and healthcare expenses of these patients. The impact and effectiveness of self-care has been observed before in patients’ medical and personal outcomes[4]. Self-care can reduce patients’ expenses, improve their quality of life, and it should be noted that its significance has been evident in patients’ medical and person-centred results [5]. The concept of self-compassion has a key role in self-care [6] .

Neff (2003/2009) was the first person to discuss the dimensions of self-compassion [7],[8-10] and divides them into three primary categories (e.i. common humanity, mindfulness, and self-kindness) and three subcategories including over-identifying, self- judgement, and feeling isolated[11,12].

It should be noted that self-compassion is affected by the values, beliefs, and culture of the society [13]. Also, self-compassion and its relation to self-care [7], especially in MS patients, is important as these patients usually suffer from this disease when they are young and cannot perform effectively in the society [14]. Therefore, further studies are needed to understand the dimensions of self-compassion in different communities. This study aimed at exploring the meaning and dimensions of self-compassion in Iranian MS patients; and based on the researcher's knowledge, no study has been conducted on this issue before.

**Methods**

**Aim**

This study aimed at exploring the meaning of self-compassion from Iranian MS patient’s perspective.

**Design**

Direct content analysis [15] was used alongside Neff’s definition of self-compassion, and all previously suggested dimensions of self-compassion proposed by Neff and other scholars, to better understand MS patients’ perception about self-compassion.

**Sample and setting**

Participants were purposefully selected from MS patients who had been diagnosed with the condition, from whose diagnoses at least 6 months had passed, and they had received at least 6 months treatment for it. The setting of the study was hospitals and communities of Zahedan and Tehran Province, Iran. Table 1 shows the characteristics of the participants **(Table1).**

After obtaining informed consent, interviews were carried out until data saturation was met. In general, 23 interviews were carried out and from the 18^th^ interview onwards, no new codes were discovered.

**Data collection**

Individual, semi-structured, in-depth face-to-face interviews were used for data gathering. Questions were designed based on Neff’s dimensions of self-compassion. The interviews lasted from 15 to 55 minutes and the average duration of each interview was 28 minutes. Participants were informed about the aim of study, with whose consent the interviews were carried out at a time and location of their own choosing – the participant’s comfortability was considered all through the interview. All interviews were conducted and recorded in Persian by the first author, some questions of which are provided hereunder:

1. From the moment you were diagnosed with MS how have you been feeling about yourself?
2. Have you ever thought about the cause of this problem? And how have you evaluated it?
3. How do you feel about yourself when you think about or see other people suffering from MS as well?

**Data Analysis**

Interviews were immediately transcribed word for word, and read several time to ensure a deep common understanding of the participant’s statements. Data was analyzed using Hsieh and Shannon’s (2005) content analysis which has three stages of preparation, organization and reporting [15]. In the preparation phase after the transcribing the interview, each text should be read several times until data is immersed. Then, in the organization stage, the researcher designs an unconstrained matrix that would allow the detection of the main concepts – or in other words, the main categories. In order to find the content that corresponds or could have corresponded to the previously defined categories, data is reviewed many times and primary codes are assigned. Here, other meaningful units are encoded which are unrelated to the main categories but related to the concept of self-compassion in general, based on the conceptual and logical relevance of which, the possibility of incorporating these categories into existing main categories of the matrix and/or the formation of new main categories is explored[16]. All the above was carried out step-by-step in this study and gathered data was analyzed by use of MAXQDA software version 10. Table 2 illustrates this data analysis matrix designed based on Neff’s theory.

**Rigor**

Lincoln and Guba’s criteria such as credibility, transferability, consistency or dependability, and confirmatory ability were considered in this study. Member and peer checking as well as prolonged engagement techniques were used. Participants were selected with maximum variation in age, gender, marital status, educational status, length of disease, severity of disease, and disease complications. After encoding the text of an interview, it was returned to the participant to ensure that both the interviewer and interviewee had the same understanding of it. Data analysis and encoding were supervised and confirmed by the research team (the second and third authors) who are will experienced in performing qualitative research and investigating MS patients as well as issues like self-compassion. The researchers documented all stages of this research, including data gathering, analysis, and encoding (into categories and subcategories) so that it would be explorable by others.

**Results**

Seventy-six primary codes were extracted. Some of these codes were placed in Neff’s suggested six categories and Freeman’s “seeking support”, while the rest were placed in the following four new categories: spiritual resilience, concern for marital life, turning into an example for others, and concealment **(Table 2).**

1. **Self-kindness**

According to Neff’s theory, self-kindness means supporting self with kindness and understanding instead of judging or reproaching self when one is in distress [11,12].

In this study, MS patients have associated it with issues like their diets, medication, self-care, self-esteem and self-worthiness.

“When I learned that I had this disease and I received the doctor’s final diagnosis, I began searching and learning about it, even on the internet, and checked to see what would make it worse or what would make it better, and what would help to control it” (p^[[1]](#footnote-1)^8)

1. **Self-judgement**

Based on Neff’s theory, self-judgement means judging and reproaching oneself when there is pain and distress, which is the opposite of self-compassion. [11,12]. In this study, MS patients have referred to it as “not caring for oneself” and “blaming self for getting ill”. These patients shared how they had become MS patients in the first place, how they have been preoccupied by the disease ever since, and how the thought had troubled them.

“I wasn’t thinking about myself at all, I was concerned about my little baby… It wasn’t such a big deal for me to cry or snob, I was more worried about my little baby” (p2).

1. **Common humanity**

Based on Neff’s theory, “common humanity” stands for perceiving one’s experiences as part of the larger human experience rather than perceiving them as experiences that would isolate them from others[11,12]. In this study, MS patients believed that it can be achieved by fostering a positive spirit in oneself and attempting to contributed or carry out responsibilities like they used to before getting sick.

“I like to be an independent individual myself, I have come here and been hospitalized, I haven’t allowed anyone to visit me like the way they visit others, or asked anyone to bring tea or to do something for me, I tell them “no” and that I can do my stuff by myself. I am an independent person and am fine”. (p 16)

1. **Isolation**

Based on Neff’s theory, “isolation” means finding oneself the only one having the disease and alone in it; this is the opposite of “common humanity”[11,12]. In this study, MS patients have described it by terms like distance, loneliness, having negative thoughts about self, and becoming dissociable.

“I am mostly depressed, I can’t stand others I don’t feel like having people over nor do I feel like going anywhere. I just stay there alone.” (p 17)

1. **Mindfulness**

Based on Neff’s theory, in the state of “mindfulness” a person observes his/her feelings and experiences as they are without magnifying them [11,12]. In this study, MS patients have associated “mindfulness” with other implications like “trying to forget being ill” , “trying to avoid thinking about the disease and its negative impacts”, “becoming adaptable and accepting the difficult treatment”, “self-construction and turning the bad feeling into good ones”, “changing one’s perspective about the disease”, “accepting the disease and coping with it” and “not blaming self because of the disease”.

“I though thinking about it would be worse, so I didn’t think about it at all… I didn’t think negatively that much as in *Hey, you are going to become paralyzed or something*….”. (p14)

1. **Over-identification**

Based on Neff’s theory, “over-identification” happens when patients magnify problems in their minds, which is the opposite of mindfulness[11,12]. In this study, in addition to the terms mentioned above, MS patients have associated “over-identification” with implications like “a paralyzed mentality” and “severe inability caused by thinking constantly about the disease and fearing it”, “unwillingness to accept the illness and inability caused by it”, “concerns about the progress of the disease by observing other patients who suffer from more advanced conditions. They expressed their concern about the progress of the disease when they encounter or had encountered patients with advanced conditions.

“I was so hopeless that I kept saying that I will die just like my sister and cousin, and I had this terrible hopeless feeling. I kept saying that I would be left alone, I would become paralyzed, I would go blind, I would get really worse… I thought about not being able to do my chores and had this really bad feeling. I kept saying I would become disabled and would need others to do my stuff… (p 22)

1. **Seeking support**

Freeman’s (2016)” seeking support” is when MS patients ask others for support[17]. In this study, MS patients described it as a “positive perspective”, “encouragement” and “supporting behaviors” that they had observed in their family members and physicians.

“My family respects me a lot and really considers my condition. The thing that MS patients hate is when people feel sorry for them. But I myself like this sympathy because I know that they love me and this is why they are doing my stuff. And this is why it didn’t feel bad, my family supported me a lot… this made me feel much better and this is why I adored their support” (p12)

With reference to the experiences of the participants, a few new categories were found as follows:

1. **Concealment**

MS patients describe it as hiding the disease from others to avoid their possible sympathy which would upset the patient or cause discomfort. Additionally, patients have also associated concealing with issues like caring for oneself and not having expectations from others.

“Sometimes someone might say something to me and try to put their kindness in words but the problem is that it is coming from their sense of sympathy, I mean they feel sorry for me, and I don’t like it”. (p9)

1. **Spiritual resilience**

MS patients associate spiritual resilience with thanking God that their illness was diagnosed early, that their illness was treatable and they had gotten better; additionally, performing religious activities, being happy and feeling fresh as the result of relying on God and believing that everything is in His will and command.

“And I thank God that I learned that I have this disease soon enough, I mean I didn’t let a year or two or more to pass …” (p2)

(p4)

1. **Marital life concern**

MS patients believe that the disease is an obstacle that is preventing them from getting married.

“I’ve seen the people around me, at my age and married, some even have kids but I have none. These moments make me regret that I didn’t get married while I was 18, I could have even had kids by now…”(p11)

1. **Turning into an example for others**

MS patients tried to share their positive experiences with others and turn into an example or a guide for others.

“After a while that I was hospitalized here, I saw this patient… He was in a very bad condition… I tried to keep his spirit high and told him that he shouldn’t be like this – because he was getting really worse and he had lost all hope. I told him a lot of these stuff like he shouldn’t lose his hope…”(p15)

**Discussion**

The purpose of this study was to explore the meaning of self-compassion in MS patients. Our findings repeated previously suggested dimensions self-compassion like self-kindness, self-judgment, common humanity, isolation, mindfulness, over-identification[11], and seeking support[17], and added four new dimensions of concealment, spiritual resilience, concern about marital life, and turning into an example for others.

Findings were in concordance with the results of Klingle (2017) who explored the perception of self-compassion among juveniles with difficult and painful life experiences. Kingle’s two implications of “Putting oneself in the center of attention” and having “a balanced emotional experience” are similar to “becoming attractive to others" and “mindfulness” in this study, respectively. Also, Reekers’ (2012) study which was conducted on social workers showed that dimensions like “gentleness”, “mindfulness” and “human connection” overlap with dimensions like “self-kindness”, “mindfulness” and “common humanity”, respectively[18]. Dimensions like “connecting with the experiences of others”, “recognizing distress”, “identifying personal accountability” and “allowing the agency of others” suggested in Freeman’s (2016) study conducted on teachers, overlap with “humanity” and “mindfulness”, respectively[17].

The results of this study are similar to those of the abovementioned studies as self-compassion is a natural, acquiring and multidimensional feature of a human[19]. Various factors such as life experiences, cultural conditions, and values [20] are effective in people’s understanding of self-compassion but compassion, satisfaction, love patience, and tolerance are all features of the human nature[21]. In this study, MS patients who have chronic conditions inherently have human nature and features despite the different socio-cultural setting that they are in or the various life experiences that they have, and Neff’s six dimensions are also applicable to them.

Concealment is a new dimension used to explore self-compassion in this study and it means that the patient tries to rely on him/herself and not seek support from others by concealing his/her disease. This dimension is the opposite of seeking support[17] which meant that one seeks the support of others during one’s illness. Acorn and Joachim (2000) showed that patients who have chronic illnesses with observable or unobservable symptoms may conceal their illness from others to avoid sympathy, stigma, and/or isolation. Also, MS patients too have observable and unobservable symptoms and the same issue applies to them as well[22].

Another finding of this study was religious resilience. MS patients try to tolerate or overcome their illness by patience, trusting in God’s will, and also performing religious activities.

Resilience is a kind of adaptation or strengthening that one has when encountering difficulties, which is achieved by interior and exterior resources. On one hand, spirituality is a way to reach adaptability and a method to facilitate the understanding of issues or intentions. Spirituality and resilience are interrelated and spiritual resilience is an ability by which one can maintain one’s feelings and intentions by a body of beliefs, values or principles. This is while, by use of interior and exterior spiritual resources, one may encounter stress, pressure and harm[23]. In Rickers’ study (2012) acceptance is a dimension which stands for accepting one’s strengths and weaknesses as well as accepting specific challenging conditions which the patient may also have no control over; and it also stands for calmness when individuals experience self-compassion[18]. In this study, MS patients by accepting their illness, by relying on God, and by being patient and calm, try to carry out their religious deeds and try to reinforce their positivity by thinking of God and accepting His will.

Pham et al.'s (2019) study on patients with chronic kidney disease showed that the meaning of transcendence, religion, church attendance, prayer and other spiritual activities, make individuals stronger and more able to reinforce their positivity, relying on self, connecting to others and God, and empowering oneself to be ready to live with a chronic disease[24].

Over 97% of the participants of this study are Muslims[25], so their beliefs and values influence their lives and behaviors[26]. In Islamic texts, dimensions like considering one’s bounty and happiness, avoiding sorrow, coping with self, having compassion for self , avoiding negligence and being fair about self, are suggested dimensions for self-compassion[27]. In this study, MS patients try to tolerate their illness by relying on spiritual activities, religion and resilience.

Another new category was being concerned about one’s marital life which was extracted from the experiences that the participants shared while explaining how they perceived self-compassion. Married patients were worried that their marriage might end, and single patients were concerned that they may never get married. When physical health is impaired in a chronic disease, it significantly affects one’s interactions with one’s partner and relatives[28].Living with a chronic progressive disorder such as MS not only causes stress for patients but also brings considerable anxiety to the patient’s dear ones. This disease affects the patient’s familial and social life, and patient may suffer from family disputes, social conflicts, divorce, etc.[29].This disease is complicated and threatening which brings a great deal of stress for one’s spouse and may lead to an unhappy marriage, separation or divorce. The results of different studies have shown that the rate of divorce is high in families where one partner is suffering from MS[30]. As expected, in this study MS patients were concerned about their marital life.

Becoming a model is another category which was extracted from the findings of this study regarding MS patients. It implied working hard to transfer one’s positive experiences and vibes to other patients and becoming their somewhat sponsor. Denis (2003) defines 'peer support' in the context of healthcare as: “providing emotional support, evaluating, and informing which are done by a member of a social network that has empirical knowledge of a certain behavior or factors that cause stress and similar issues to the target population”[31].

In Freeman’s (2016) studies, relating positively with others' experiences is a dimension of self-compassion; and this study has also shown the same. Here, MS patients try to help other patients who are suffering from a similar condition to overcome or tolerate the disease, by sharing their own relevant positive experiences. This would help others to better understand the challenges that they encounter, while simultaneously express self-compassion towards themselves[32].

**Conclusion:**

Given that Neff’s study was conducted on healthy individuals who had not suffered from difficult conditions, the dimensions that were found in this study did not surface in Neff’s. For MS patients however, who have suffered challenging conditions and have different experiences, self-compassion has a different range of dimensions. The study results can be used for improving clinical patient care, promoting self-care among ms patients.

**Funding**

None.

**References**

1. Abedini E, Ghanbari Hashem Abadi BA, Talebian-Sharif J (2016) Effectiveness of group therapy based on hope approach on hope and depression in women with multiple sclerosis. Journal of Clinical Psychology 8 (2(30).):1-11.

2. Dahmardeh H, Vagharseyyedin SA, Amiri Fard H, Sharif-zadeh GR, Rakhshani-Zabol F (2015) Effect of self-care educational program based on Orem’s Theory on hope in patients with Multiple Sclerosis. Medical - Surgical Nursing Journal 4 (2):57-63

3. Karussis D (2014) The diagnosis of multiple sclerosis and the various related demyelinating syndromes: A critical review. Journal of Autoimmunity 48 (49):134-142

4. Jaarsma T, Cameron J, Riegel B, Stromberg A (2017) Factors Related to Self-Care in Heart Failure Patients According to the Middle-Range Theory of Self-Care of Chronic Illness: a Literature Update. Curr Heart Fail Rep 14:71–77

5. Wilkinson A, Whitehead L (2009) Evolution of the concept of self-care and implications for nurses: A literature review. International Journal of Nursing Studies 46:1143–1147

6. Sinclair S, Kondejewski J, Raffin-Bouchal S, M. King-Shier K, Singh P (2017) Can Self-Compassion Promote Healthcare Provider Well-Being and Compassionate Care to Others? Results of a Systematic Review. Applied psychology: health and well-being:1-39

7. Ferrari M, Dal Cin M, Steele M (2017) Educational and psychological aspects Self-compassion is associated with optimum self care behaviour, medical outcomes and psychological well-being in a cross-sectional sample of adults with diabetes. Diabetic Medicine 34 (11)

8. Nery-Hurwit M, Yun J, Ebbeck V (2017) Examining the roles of self-compassion and resilience on health-related quality of life for individuals with Multiple Sclerosis. Disability and Health Journal 1-18

9. Amanullah A, Tirdat K, Aslani K (2016) Prediction of Depression Based on Components of self- Compassion in Girl Students with Emotional Breakdown Experience in Ahvaz Universities. Journal of Clinical Psychology 8 (2):77-87

10. Moszadeh S (2017) Effectiveness of self-directed group training on reducing the stress of parenting and increasing parental self-efficacy and resilience of mothers of children with autism spectrum disorder. Mashhad Ferdowsi University,

11. Neff KD (2003) Self-Compassion: An Alternative Conceptualization of a HealthyAttitudeTowardOneself. Self and Identity 2:85–101

12. Raes F, Pommier E, Neff KD, Van Gucht D (2011) Construction and Factorial Validation of a Short Form of the Self-Compassion Scale. Clinical Psychology and Psychotherapy 18:250-255

13. Neff KD, Pisitsungkagarn K, Hsieh Y (2008) Self-compassion and self-construal in the united states, thailand, and taiwan. Journal of cross-cultural psychology 39 (3):267-285

14. Reyes D (2012) Self-Compassion:A Concept Analysis. Journal of Holistic Nursing American Holistic Nurses Association 30 (2):81-89

15. Hsieh HF SS (2005) Three approach to qualitative content analysis. . Qualitative health research 15 (9):1277-1288

16. Elo S, Kyngas H (2008) The qualitative content analysis process. J Adv Nurs 62 (1):107-115.

17. Freeman S (2016) Emotions in Teaching: Self-Compassion. Brigham Young University,

18. Rickers S (2012) The Lived Experience of Self-Compassion in Social Workers. The university of minnesota,

19. Montero-Marin J, Kuyken W, Crane C, Gu J, Baer R, A. Al-Awamleh A, Akutsu S, Araya-Véliz C, Ghorbani N, Job Chen Z, Kim MS, Mantzios M, N. Rolim dos Santos D, C. Serramo López L, A. Teleb A, Watson PJ, Yamaguchi A, Yang E, García-Campayo J (2018) Self-Compassion and Cultural Values: A Cross-Cultural Study of Self-Compassion Using a Multitrait-Multimethod (MTMM) Analytical Procedure. Frontiers in Psychology 9

20. McGehee P, Germer C, Neff KD (2017) Core Values in Mindful Self-Compassion. In: Practitioner's Guide to Ethics and Mindfulness-Based Interventions. pp 279-293. doi:DOI: 10.1007/978-3-319-64924-5_11

21. Neff KD, Rude SS, Kirkpatrick K (2007) An examination of self compassion in relation to positive psychological functioning and personality traits. Journal of Research in Personality 41 (4):909

22. Joachim G, Acorn S (2000) Stigma of visible and invisible chronic conditions. Journal of Advanced Nursing 32 (1):243-248. doi:doi:10.1046/j.1365-2648.2000.01466.x

23. Manning L, Ferris M, Rosario CN, Prues M, Bouchard L (2018) Spiritual resilience: Understanding the protection and promotion of well-being in the later life. Journal of Religion, Spirituality & Aging:1-20

24. Tony VP, Cherry MB, Jane PG, Harold GK, John WS (2019) Spirituality, Coping, and Resilience Among Rural Residents Living with Chronic Kidney Disease. Journal of Religion and Health:1-18

25. Fathi E (2016) A look at the religion and population of iran in the half a century. Statistics 21 (December and January )

26. Yaghob F (2015) Demographic Survey of Muslims in Contemporary World. Islamic World Political Research Quarterly 3 (5):61-98

27. Zinali R (2016) Compassion for oneself in Islamic teachings. mishkat 132 (Fall):38-60

28. Alavian SM, Kachuee H, Moghani Lankarani M, Assari S, Farmanara H (2006) Marital adjustment in patients with chronic viral hepatitis versus healthy controls. . Iran J psychiatry 1 (4):103-107.

29. Sadat SJ, Alimohammadi N, Alamdary AK (2011) Phenomenological study of family relationships and Social patients with multiple sclerosis. Journal of Mazandaran University of Medical Sciences 21 (1):244-202.

30. Tajikesmaeili A, Hakim abadi MG (2016) Sexual functions and marital adjustment married woman with Multiple Sclerosis. Journal of Research in Psychological Health 10 (2):1-10

31. Dennis CL (2003) Peer support within a health care context: a concept analysis. International Journal of Nursing Studies 40 (321-32)

32. Ng L, Amatya B, Khan F (2013) Outcomes of a Peer Support Program in Multiple Sclerosis in an Australian Community Cohort: A Prospective Study. Journal of Neurodegenerative Diseases 1:1-7. doi:DOI: 10.1155/2013/429171

T

Table 1. Characteristics of study participants

| Gender | | Marital status | | Educational status | | Duration of illness (year) | | Different levels of severity of disease and its complications | |
| --- | --- | --- | --- | --- | --- | --- | --- | --- | --- |
| Data | Variable | Data | Variable | Data | Variable | Data | Variable | Data | Variable |
| 20 | Female | 7 | Single | 1 | Illiterate | 2 | Six months-1year | 12 | Eye problems (poor eyesight) |
| 3 | Male | 13 | Married | 6 | High school | 6 | 1-5 years | 13 | Difficult walking (walking disorder) |
|  |  | 3 | Divorced | 6 | Diploma | 10 | 5-10 years | 4 | Urinary and bowel problem |
|  |  |  |  | 9 | Bachelor’s degree | 4 | 10-15 years | 23 | Fatigue |
|  |  |  |  | 1 | Master’s Degree | 1 | 15-20 years | 2 | Limb paralysis |
|  |  |  |  |  |  |  |  | 13 | Muscle Weakness |

Table 2. Data analysis matrix based on Neff’s dimension

| **Primary codes** | | **Categories based on Neff’s dimension** |  |
| --- | --- | --- | --- |
| - Carrying for the proper consumption of medicine, despite the fact that it one might not consider it to be exclusory - Relying on medication and proper nutrition for self and family - Relying on medication for one’s family - Following-up on the treatment - Feeling more confident at home than in society - Feeling more confident and valuable - Emphasizing confidence, self-esteem, optimality and empowerment - Paying more attention to one’s health and self-care - Trying to avoid stressful and uncomfortable situations - Putting oneself at the center of attention - Reaching a sense of self love and self-respect | | **Self-kindness** |  |
| - Not respecting self - Caring more for others and less for self - Not taking one’s illness seriously - Blaming self and one’s former behavior for getting ill - Feeling insignificant to others - Mindfulness (an occupied mind) about why one is now suffering from the illness | | **Self-judgement** |  |
| - Trying to induce the feeling of hope to the effectively of treatment and reinforcing positivity in self - Trying to show oneself positive and normal - Trying to develop a positive perspective towards the weaknesses that have been caused - Trying to rely on self - Emphasizing patience and tolerance to overcome the illness - Trying to perform social role (studying and exercising) just like one did before getting ill - Trying to represent self like a healthy person - Developing a good feeling/hope in self by observing/speaking with other patients who suffer from the same illness - Developing a feeling that one is good and better when one compares one’s condition with that of other patients | | **Common humanity** |  |
| - Avoiding others - Having negative thoughts about self and becoming isolated - Willing to be distant and alone | | **Isolation** |  |
| **Primary codes** | | **Categories based on Neff’s dimension** |  |
| - Trying to forget and not think about the illness - Concealing the illness from one’s family to maintain one’s health and comfort - Not believing that the illness is an obstacle to living - Avoiding thinking about the illness and its negative aspects - Accepting the illness and coping with it - Accepting the illness and at the same time relying on God - Not blaming self about the illness - Observing the illness as a simple issue - Trying to overcome symptoms that affect the senses, though simple ways - Emphasizing optimism and proudness when the symptoms of the illness no longer reappear - Emphasizing the effort to overcome the symptoms that negatively affect the illness - Not feeling bad because of not knowing the nature of the illness - Converting bad feelings about the illness to good ones - Changing one’s perspective towards the illness | | **Mindfulness** |  |
| - Doing less activities because of the illness - To attribute the emergence of new plaques to their coping - Not willing to accept the illness and the inability that follows - The thought and concern about the progress of the illness by observing other patients suffering from advance stages of the illness - Fear of paralysis - Thinking a lot about paralysis - Mental paralysis and severe inability as the result of the illness | | **Over- identification** |  |
| - Being happy about what one has and being thankful for one’s family support - Reaching a feeling of calmness when there is family and doctor’s support, trying to be positive and perform effectively - Being positive about the consideration, thoughtfulness and support of others - Feeling better and more positive with the support, words, or help of the doctor | | **Seeking support** |  |
|  | | | |
| **Remaining codes that could not be** | | | |
| **Primary code** | **New category** | | |
| - Concealing the illness from others to avoid others’ pity - Not wanting or needing the pity of family or others - Not wanting others’ attention - Being upset if others perform one’s tasks - Thinking about self and not expecting from others | **Concealment (the negative dimension of support seeking)** | | |
|  |  | | |
| **Primary code** | **New category** | | |
| - Understanding and being thankful that the illness is treatable - Thanking God that one’s condition is getting better - Thanking God that that the illness is diagnosed - Thanking God when comparing one’s condition with that of others whose condition is worse - Reinforcing calmness and positivism with believing in the illness - Knowing that the illness is a God’s way of testing one - Becoming calm when performing religious activities - Not committing suicide for fearing God - Feeling happy and fresh when relying on God - Emphasizing on the religious explanation of becoming ill, and being patient | **Religious resilience** | | |
| - Concern about not getting married - Concern about the future of a martial life | **Concern about martial life** | | |
| - Trying to become a model or guide for others while also carrying for one’s health - Becoming a model for others - Transferring one’s positive feelings and experiences to others | **Becoming a role model for others** | | |

1. Patient [↑](#footnote-ref-1)
